# Effects of Root–Root Interactions on the Physiological Characteristics of *Haloxylon ammodendron* Seedlings

**DOI:** 10.3390/plants13050683

**Published:** 2024-02-28

**Authors:** Huifang Yang, Suwan Ji, Deyan Wu, Menghao Zhu, Guanghui Lv

**Affiliations:** 1 College of Ecology and Environment, Xinjiang University, Urumqi 830017, China; 107552101166@stu.xju.edu.cn (H.Y.); jsw@stu.xju.edu.cn (S.J.); bingyeshifu@163.com (D.W.); zhumenghao1999@163.com (M.Z.); 2 Key Laboratory of Oasis Ecology of Education Ministry, Xinjiang University, Urumqi 830017, China; 3 Xinjiang Jinghe Observation and Research Station of Temperate Desert Ecosystem, Ministry of Education, Urumqi 830017, China

**Keywords:** root–root interactions, functional traits, root metabolites, soil bacteria, soil nutrients, *Haloxylon ammodendron*

## Abstract

The root traits and response strategies of plants play crucial roles in mediating interactions between plant root systems. Current research on the role of root exudates as underground chemical signals mediating these interactions has focused mainly on crops, with less attention given to desert plants in arid regions. In this study, we focused on the typical desert plant *Haloxylon ammodendron* and conducted a pot experiment using three root isolation methods (plastic film separation, nylon mesh separation, and no separation). We found that (1) as the degree of isolation increased, plant biomass significantly increased (*p* < 0.05), while root organic carbon content exhibited the opposite trend; (2) soil electrical conductivity (EC), soil total nitrogen (STN), soil total phosphorus (STP), and soil organic carbon (SOC) were significantly greater in the plastic film and nylon mesh separation treatments than in the no separation treatment (*p* < 0.05), and the abundance of Proteobacteria and Actinobacteriota was significantly greater in the plastic film separation treatment than in the no separation treatment (*p* < 0.05); (3) both plastic film and nylon mesh separations increased the secretion of alkaloids derived from tryptophan and phenylalanine in the plant root system compared with that in the no separation treatment; and (4) Pseudomonas, Proteobacteria, sesquiterpenes, triterpenes, and coumarins showed positive correlations, while both pseudomonas and proteobacteria were significantly positively correlated with soil EC, STN, STP, and SOC (*p* < 0.05). Aurachin D was negatively correlated with Gemmatimonadota and Proteobacteria, and both were significantly correlated with soil pH, EC, STN, STP, and SOC. The present study revealed strong negative interactions between the root systems of *H. ammodendron* seedlings, in which sesquiterpenoids, triterpenoids, coumarins, and alkaloids released by the roots played an important role in the subterranean competitive relationship. This study provides a deeper understanding of intraspecific interactions in the desert plant *H. ammodendron* and offers some guidance for future cultivation of this species in the northwestern region of China.

## 1. Introduction

Plant–plant interactions are complex processes, particularly in the rhizosphere, where plants can sense and recognize coexisting conspecific or heterospecific plants, thereby adjusting their growth, reproduction, and defense strategies [1,2,3]. The rhizosphere not only serves as a direct interface for energy flow and material exchange between plant roots and the soil but also represents a complex and highly dynamic environment for interactions among plant roots, soil pathogens, beneficial microorganisms, invertebrates, and competitors, making it one of the most active interfaces on Earth [4,5,6]. As direct mediators of plant interactions with this dynamic environment, root secretions play a crucial role in transmitting information and facilitating active adaptation and defense against various adverse conditions in plants [7,8].

Plant roots secrete a diverse array of compounds known as root exudates into the surrounding soil. These exudates consist of various substances that are released or emitted from different parts of the root system during plant growth. They include primary metabolites such as sugars, amino acids, and organic acids as well as secondary metabolites including phenolics, alkaloids, flavonoids, and terpenes [9,10,11,12,13]. Primary metabolites provide the substances and energy necessary for plant growth and development, as well as for the growth and development of herbivores and microorganisms [14] (Koch, 2004), while secondary metabolites mainly regulate the interactions between plants and other organisms, as well as the environment [15,16]. On the one hand, root metabolites can improve the ability of plant roots to absorb and utilize nutrients and adapt to changes in the external environment by altering the mechanical and biochemical properties of the rhizosphere. Several root exudates are known to promote the availability of soil nutrients [17,18]. For example, the concentration of available ions such as P and Zn increases with the exudation of acid phosphatase, protons, and/or carboxylates [19]. Recent studies have also demonstrated that root exudates can accelerate organic matter mineralization (priming effect), where exudates such as amino acids, sugars, and flavonoids mediate soil nitrogen mineralization by influencing the soil environment [20]. The various effects of root exudates can impact the nitrogen and phosphorus cycles in the rhizosphere, benefiting plants [21,22]. The role of root secretions extends beyond increasing nutrient availability: they also directly influence the outcomes of root–root interactions in the rhizosphere [7]. One of the most significant effects is allelopathy, in which plants release allelochemicals that reduce the establishment, growth, or survival of neighboring plants, thereby reducing competition and increasing resource availability. These allelochemicals include diverse types and structures, including phenolics (such as phenolic acids and flavonoids), terpenoids, alkaloids, and other secondary metabolites [23,24,25]. Some of these allelochemicals, such as phenolics and acid substances, aid in attracting nutrients such as N, P, and metal ions, enhancing plant competitiveness and indirectly inhibiting neighboring plants. On the other hand, root exudates exert a pivotal influence on the composition and dynamics of the microbial community inhabiting the rhizosphere [26], serving as key regulators of rhizosphere microbial ecological functions. Released compounds have been shown to attract beneficial microorganisms and influence the assembly of rhizosphere microbiota, thereby enhancing plant adaptation to the environment. Sesquiterpenes in root exudates have been found to regulate the abundance of actinobacteria [27], phenolic substances act as key chemical inducers of rhizobacterial growth promotion and defense against pathogens [28,29], and benzoxazinoids in maize root exudates attract bacteria from the phylum Chloroflexi and influence root microbiota assembly, ultimately enhancing the environmental adaptability of maize plants [30]. The above research indicates that root exudates have a significant impact on the construction and composition of microbial communities.

It is well established that compounds secreted by plant roots play an important role in underground interactions among plants, by affecting the soil environment and rhizosphere microorganisms. However, most of the related research has focused on crops, with little attention given to desert woody plants in arid regions. *H. ammodendron*, a small deciduous tree in the Chenopodiaceae family, is a dominant species in plant communities in northwestern China’s deserts. This study aims to investigate whether the role of root secretions is stronger than root contact in belowground interactions among *H. ammodendron* seedlings. We also explored how inter-root interactions affect nutrient utilization, microbial environments, and plant growth. This study aims to identify the compounds that play a significant role in these interactions, to reveal the chemical relationships within plant species. Based on this, we hypothesize that under the competitive relationship between *H. ammodendron* seedlings, the compounds released by the roots play a stronger role in belowground interactions among *H. ammodendron* seedlings. This research is not only significant for understanding the mechanisms underlying plant interactions and coexistence in ecological systems but also for promoting afforestation and vegetation restoration in China’s northwestern desert region.

## 2. Results

### 2.1. Effect of Root Separation on the Physiological Characteristics of H. ammodendron Seedlings

**Figure 1:**
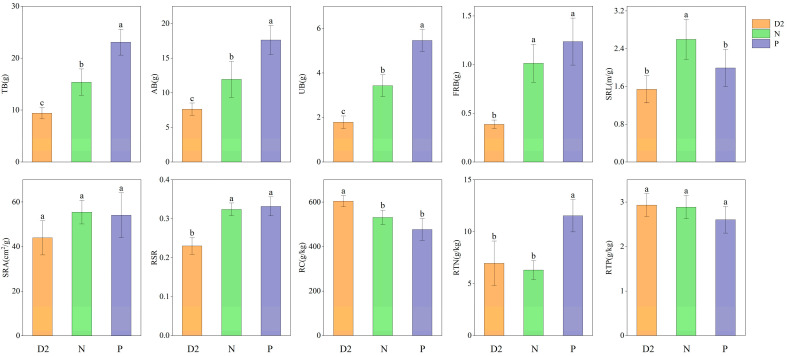
Biomass and root characteristics of *H. ammodendron* seedlings from different separation methods. Different letters indicate significant differences between treatments (*p* < 0.05): TB: total biomass; AB: aboveground biomass; UB: underground biomass; FRB: fine root biomass; SRL: specific root length; SRA: specific leaf area; RSR: root-shoot ratio; RC: root organic carbon; RTN: root total nitrogen; RTP: root total phosphorus; D2: no separation group; N: nylon net separation group; P: plastic film separation group.

There were significant changes in the morphological and structural characteristics of *H. ammodendron* seedlings under the different separation methods (Figure 1). As the degree of separation increased, the total biomass (TB), aboveground biomass (AB), and underground biomass (UB) content significantly increased, with plastic film separation > nylon net separation > no separation. The FRB content under the plastic film separation and nylon mesh separation treatments was significantly higher than that of the nonseparated treatment (*p* < 0.05), but there was no significant difference between the two separation methods (*p* > 0.05). The specific root length (SRL) under nylon net separation was significantly higher than the other two groups (*p* < 0.05); the specific leaf area (SRA) and root-shoot ratio (RSR) were significantly higher under nylon net and plastic film separation than under no separation treatment (*p* < 0.05); the root organic carbon (RC) was significantly higher under no separation than under separation treatments (*p* < 0.05), showing no separation > nylon net separation > plastic film separation; and the root total nitrogen (RTN) was found to be highest in the group where plastic film separation was used. This particular group exhibited significantly higher RTN levels compared to the other two groups (*p* < 0.05). Nevertheless, the levels of root total phosphorus (RTP) were not observed to be significantly affected by the different treatments (*p* > 0.05).

### 2.2. Soil Physicochemical Properties and Bacterial Community Structure Characteristics of H. ammodendron Seedlings under Different Separation Methods

**Figure 2:**
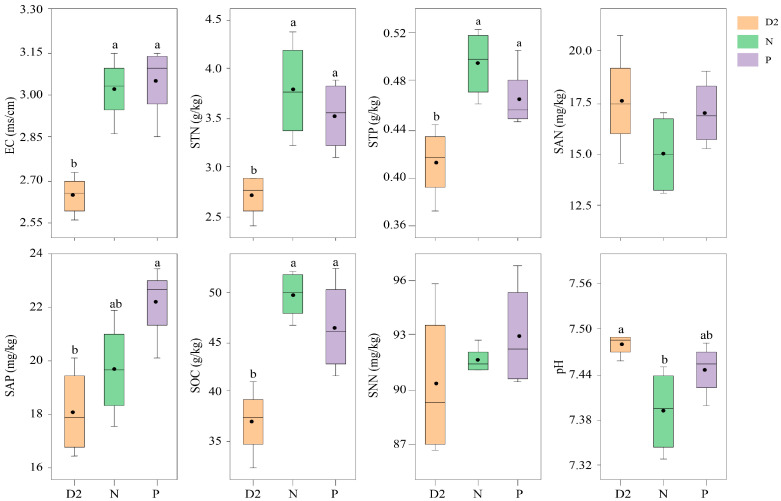
Variations in rhizosphere soil physicochemical properties were examined under different separation conditions. Significant differences (*p* < 0.05) among the different treatments are denoted by different letters, while treatments without letters indicate nonsignificant differences between groups: SOC: soil organic carbon; STN: soil total nitrogen; SAP: soil available phosphorus; STP: soil total phosphorus; EC: electrical conductivity; pH: soil pH; SNN: soil nitrate nitrogen; SAN: soil ammonium nitrogen; D2: no separation treatment; N: nylon net separation treatment; P: plastic film separation treatment.

According to Figure 2, the separation methods significantly influenced the physicochemical properties of the rhizosphere soil of *H. ammodendron* seedlings. The soil electrical conductivity (EC), total nitrogen (STN), total phosphorus (STP), and soil organic carbon (SOC) contents were significantly greater in the plastic film and nylon net separation treatments than in the no separation treatment (*p* < 0.05); however, there was no significant difference between the plastic film and nylon net separation treatments (*p* > 0.05). The soil available phosphorus (SAP) content gradually increased with increasing separation degree and was significantly greater in the plastic film separation treatment than in the no separation treatment (*p* < 0.05). The soil pH decreased first and then increased with increasing separation degree, with the highest pH observed in the no separation treatment, which was significantly greater than that in the nylon net separation treatment (*p* < 0.05). The different separation treatments had no significant effect on the soil ammonium nitrogen (SAN) or soil nitrate nitrogen (SNN) contents.

The results of the correlation analysis of the soil physicochemical properties of the rhizosphere soil of *H. ammodendron* seedlings are shown in Table 1. There was a significant positive correlation (*p* < 0.05) between EC and SOC, as well as a highly significant positive correlation (*p* < 0.01) between EC and STN. Soil pH exhibited a highly significant negative correlation (*p* < 0.01) with SOC and STN and a significant negative correlation (*p* < 0.05) with STP. On the other hand, there was a highly significant positive correlation (*p* < 0.01) between SOC and STN and between SOC and STN. Furthermore, a significant positive correlation (*p* < 0.05) was observed between the STP and STN.

**Figure 3:**
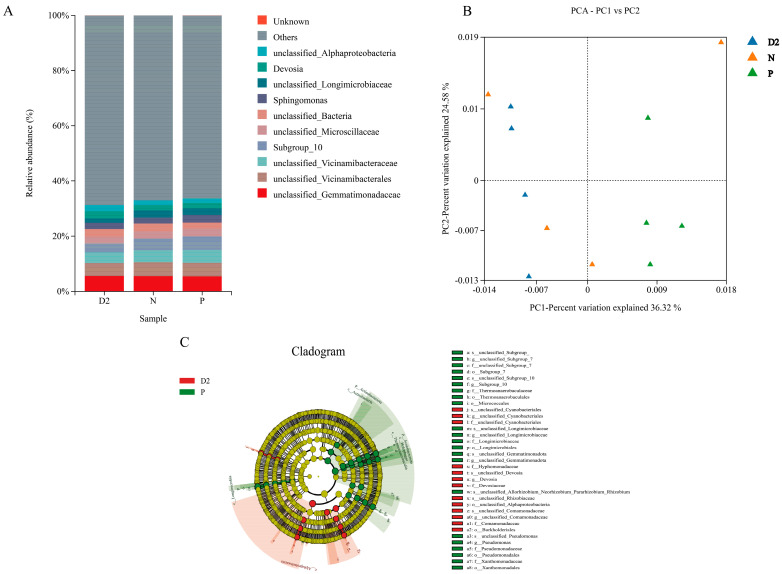
Genus-level bacterial community composition and distribution under different isolation methods: (**A**) A bar chart illustrating the distribution of bacterial taxa in terms of their relative abundance. (**B**) Principal component analysis (PCA) plot of the bacterial community composition. (**C**) LEFSe analysis. D2: Nonisolation group; N: Nylon net isolation group; P: Plastic film isolation group.

16S V3-V4 region sequencing was carried out to investigate the microbial diversity in the rhizosphere soil of *H. ammodendron* seedlings under different isolation methods. A total of 38 phyla, 108 classes, 285 orders, 557 families, 1004 genera, and 1176 species were detected. The dilution curve and Shannon curve tended to flatten, and the coverage rate was greater than 98%, indicating that the sequencing data were sufficient for data analysis. At the genus level, the top 10 abundant taxa were *unclassified_Gemmatimonadaceae* (Gemmatimonadetes), *unclassified_Vicinamibacterales* (Acidobacteria), *unclassified_Vicinamibacteraceae* (Acidobacteria), *Subgroup_10* (Acidobacteria), *unclassified_Microscillaceae* (Planctomycetes), *unclassified_Bacteria* (Unknown), *Sphingomonas* (Alphaproteobacteria), *unclassified_Longimicrobiaceae* (Gemmatimonadetes), *Devosia* (Alphaproteobacteria), and *unclassified_Alphaproteobacteria* (Alphaproteobacteria) (Figure 3A). As shown in Figure 3B, PCA revealed significant changes in the bacterial community composition between the nonisolation treatment and plastic film isolation treatment groups, which could be separated along the first coordinate axis. However, there was no significant change in community composition between the nonisolation treatment and the nylon net isolation treatment. LEFSe differential analysis revealed significant differences (LDA > 3.5) in the abundance of Proteobacteria and Actinobacteria at the phylum level and *Pseudomonas* and *Subgroup_10* at the genus level in the plastic film isolation treatment compared to the nonisolation treatment. Additionally, *unclassified_Cyanbacteriales*, *Devosia*, and *unclassified_Comamonadaceae* were significantly enriched in the nonisolation treatment (Figure 3C).

### 2.3. Metabolite Composition and Quantitative Characteristics of H. ammodendron Seedling Roots under Different Isolation Methods

**Figure A1:**
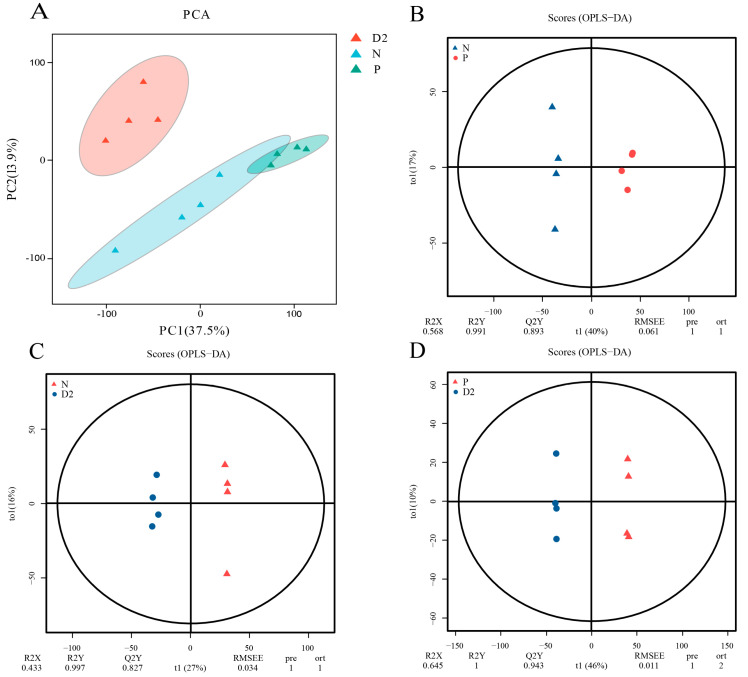
PCA and OPLS-DA of root metabolites in *H. ammodendron* seedlings under different separation methods. (**A**): PCA analysis of root secretion *H. ammodendron* seedlings under different separation treatments. (**B**): OPLD-DA analysis of root secretions of *H. ammodendron* seedlings between N and P treatments. (**C**): OPLD-DA analysis of root secretions of *H. ammodendron* seedlings between N and D2 treatments. (**D**): OPLD-DA analysis of root secretions *H. ammodendron* seedlings between P and D2 treatments.

LC-MS was used for metabolomic analysis of the root secretions of *H. ammodendron* seedlings, and a total of 4010 metabolites were identified. Based on the KEGG class classification, these metabolites were divided into 15 root metabolite types (Table 2), mainly including lignans, neolignans, and related compounds (13.92%); organic acids and derivatives (7.31%); organoheterocyclic compounds (5.91%); phenylpropanoids and polyketides (5.66%); organic oxygen compounds (4.91%); and benzenoids (4.66%). The PCA results (Figure A1) revealed distinct differences in the first and second principal components between the root secretions of the nonisolation treatment and plastic film isolation treatment groups, while the second principal component was separated in the nonisolation and nylon net isolation treatment groups.

Orthogonal partial least squares discriminant analysis (OPLS-DA) was performed to analyze the differences between the D2, N, and P groups pairwise (Figure A1). The results showed that the root metabolites in *H. ammodendron* seedlings, influenced by the isolation methods, exhibited stable and reliable differences in intraspecies competition (R_2_Y > 0.99, Q2Y > 0.85, and R2Y > Q2Y). Based on the variable importance in projection (VIP) combined with the p value from univariate analysis and fold change (FC) values, differentially abundant metabolites were further selected (FC > 2, VIP > 1, and *p* < 0.05). There were 385 (upregulated: 301; downregulated: 57), 1182 (upregulated: 916; downregulated: 266), and 824 (upregulated: 678; downregulated: 146) differentially abundant metabolites between D2 and N, D2 vs. P, and N vs. P, respectively (Table A1). The number of differentially abundant metabolites between the plastic film isolation and nylon net isolation or nonisolation methods was significantly greater than that between the nylon net isolation and nonisolation methods.

Using KEGG compound classification, the differentially abundant metabolites between the D2 and P groups were categorized as plant secondary metabolites (Table 3). A total of 51 compounds were identified (45 upregulated; 6 downregulated), which were mainly alkaloids derived from tryptophan and anthranilic acid (8 compounds), monoterpenoids (6 compounds), triterpenoids (5 compounds), monolignols (4 compounds), and flavonoids (4 compounds). Between the D2 and N groups, 17 compounds were identified (15 upregulated; 2 downregulated), mainly consisting of alkaloids derived from tryptophan and anthranilic acid (3 compounds), sesquiterpenoids (2 compounds), and isoflavonoids (2 compounds). Among the N and P groups, 21 compounds were identified (17 upregulated; 4 downregulated), mainly monoterpenoids (3 compounds) and monolignols (3 compounds).

### 2.4. Relationships between Root Metabolites and Rhizosphere Bacteria and between Root Metabolites and Soil Physicochemical Properties

**Figure 4:**
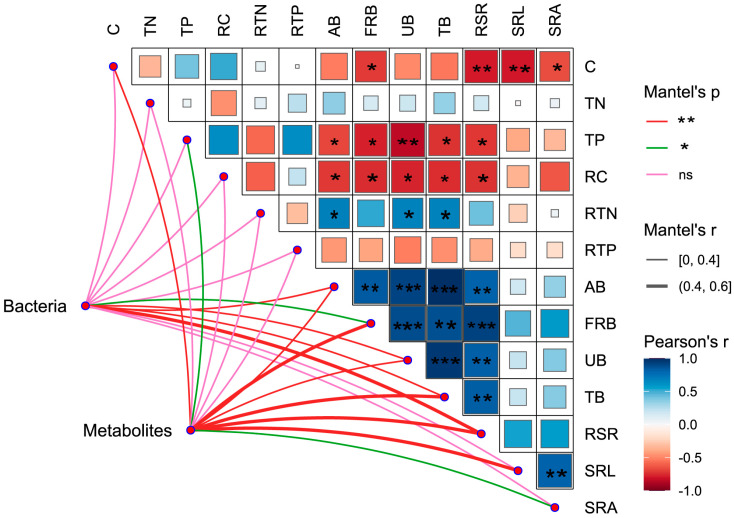
Correlations among plant traits and Mantel test results for bacterial and root exudate relationships: C: aboveground plant organic carbon; TN: aboveground plant total nitrogen; TP: aboveground plant total phosphorus. For the meanings of the remaining letters, please refer to Figure 1. *: *p* ≤ 0.05; **: *p* ≤ 0.01; ***: *p* ≤ 0.001; ns: insignificant; Spearman correlation.

Under the different isolation methods, there were strong positive correlations among AB, UB, TB, FRB, RSR, and SRL. The total phosphorus (TP) in the aboveground plant (TP) community was positively correlated with the RC and RTP. RC exhibited a negative correlation with RTN. There were no significant correlations observed between the other nutrient traits. The aboveground plant organic carbon (C) and total protein (TP) contents were negatively correlated with the various morphological traits of the plants (Figure 4). The Mantel test was used to examine the relationships between bacterial communities, root exudates, and plant traits. There were 13 significant relationships, including a significant relationship between bacterial communities and FRB (*p* < 0.05) and extremely significant relationships between bacterial communities and UB, TB, and RSR (*p* < 0.05). Significant relationships were also detected between plant root exudates and TP and specific root area (SRA) (*p* < 0.05), as well as between plant root exudates and C, AB, FRB, UB, TB, RSR, and SRL (*p* < 0.01) (Figure 4).

**Figure 5:**
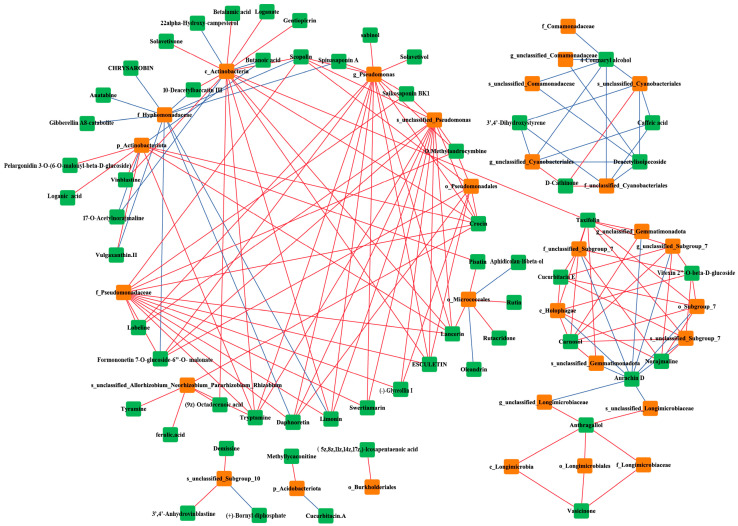
Correlation analysis between differential secondary metabolites and differential bacterial groups. The red lines represent positive correlations; the blue lines represent negative correlations. Green boxes represent differential flora and orange represents differential metabolites.

Based on *p* ≤ 0.001 and |R| ≥ 0.85, 30 different bacterial groups and 58 different metabolites were strongly correlated, resulting in a total of 336 correlated relationships (Figure 5). In addition, Hyphomonadaceae was negatively correlated with diterpenes, triterpenes, and other compounds, while Pseudomonas, Pseudomonadaceae, Proteobacteria, and other groups were positively correlated with sesquiterpenes, triterpenes, and coumarins. Similarly, the abundances of the Comamonadaceae, Cyanobacteriales, and other groups were negatively correlated with the abundance of Deacetylisoipecoside, an alkaloid. Aurachin D was found to be negatively correlated with Gram-negative bacteria such as Gemmatimonadota and Proteobacteria.

**Figure 6:**
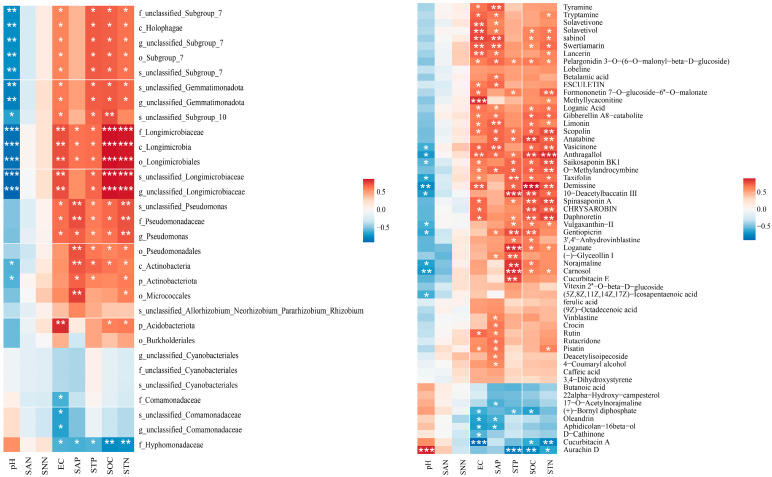
Correlation analysis between differential bacterial groups, differential secondary metabolites, and soil environmental factors: **left**: correlation analysis between differential bacterial groups and soil physicochemical properties; **right**: correlation analysis between differential secondary metabolites and soil physicochemical properties. The meanings of the letters can be found in Figure 2. *: *p* ≤ 0.05; **: *p* ≤ 0.01; ***: *p* ≤ 0.001.

A correlation analysis was performed between the selected differential bacterial groups and metabolites and the physicochemical properties of the soil (Figure 6). Overall, there were significant correlations between the soil physicochemical properties and both the differential bacterial groups and metabolites. There was a distinct negative correlation between the different bacterial groups and pH, while a strong positive correlation was found with soil organic carbon, total phosphorus, and total nitrogen. The differentially abundant metabolites exhibited some variation. In detail, in the left panel, the phylum Gemmatimonadota, specifically the class Longimicrobia, was most affected by soil conditions, particularly soil pH, electrical conductivity, and soil carbon and phosphorus. Additionally, the phylum Acidobacteriota, specifically the class Holophagae; the phylum Actinobacteria, specifically the order Pseudomonadales; and Hyphomonadaceae were also influenced by the soil environment. In the right panel, compounds such as saikosaponin BK1, spinasaponin A, anthragallol, and CHRYSAROBIN, which are triterpenes, exhibited strong positive correlations with soil organic carbon, available phosphorus, total phosphorus, and total nitrogen. Flavonoids were also positively correlated. Conversely, the alkaloid aurachin D exhibited a strong negative correlation with soil carbon, nitrogen, and phosphorus. In conclusion, the root exudates of *H. ammodendron* seedlings are closely related to plant growth, soil nutrients, and rhizosphere bacteria, especially triterpenes, sesquiterpenes, diterpenes, and alkaloids.

## 3. Discussion

### 3.1. Effects of Root Contact and Secretions on the Physiological Traits of H. ammodendron Seedlings

The results of this study showed that the biomass of plants with separated root systems was significantly greater than that of plants without separated root systems, and the biomass increased with the degree of separation, indicating strong competition among *H. ammodendron* salsa individuals, which is consistent with the results of Cahill et al. (2010) [31]. The plants under nylon mesh separation had significantly longer roots and larger root surface areas than did those in the nonseparated and completely separated groups, suggesting that plants under this treatment had more fine roots and a greater root surface area [32], which indicates a greater area for nutrient and water exchange between the roots and soil, promoting the absorption of nutrients and water and enhancing resource availability and competitive ability [33]. A comparison of the aboveground and belowground traits of plants under different treatments revealed that the effects of root secretions mediated by underground interactions and direct root contact on plant biomass and root characteristics varied. Direct contact had a more pronounced effect on plant biomass, while secretion-mediated effects had a more significant impact on root characteristics. This finding is consistent with the results of barrier experiments between *Allium cepa* L. var. *multiplans Baileysyn.* var. *Agrogatum Don* and *Solanum lycopersicum* roots [34]. The root-to-shoot ratio in the non-plasma-separated group was significantly lower than that in the other two groups, while the organic carbon and total phosphorus contents in the plant roots were greater than those in the other two groups. This indicates that direct root contact has a strong inhibitory effect and has a greater influence on the physiological traits of roots, such as organic carbon and total phosphorus content.

### 3.2. Root Interactions Alter the Soil Physicochemical Properties and Bacterial Community Structure of H. ammodendron Seedlings

This study showed that the contents of organic carbon, total nitrogen, and total phosphorus in the soil were the highest in the nylon mesh-separated group, followed by those in the plastic film-separated group, both of which were significantly greater than those in the nonseparated group. Earlier studies have suggested that root secretions, which include organic acids, have a significant impact on soil development by enhancing the dissolution and movement of phosphorus. Additionally, these secretions can modify soil physicochemical characteristics, thereby facilitating the uptake of essential nutrients by plants [35,36]. The isolation of underground root systems from the same plant species reduces the degree of competition between plants and may also alleviate microbial competition in organic matter decomposition processes, leading to the retention of more organic carbon, total nitrogen, and total phosphorus in the soil. Previous studies have shown positive correlations between carbon, nitrogen, phosphorus, and electrical conductivity [37], which also explains why the soil electrical conductivity was significantly greater in the nylon mesh-separated and plastic film-separated treatments than in the nonseparated root system treatment.

The dominant bacterial groups in the rhizosphere soil of *H. ammodendron* seedlings mainly consisted of Proteobacteria, Acidobacteria, Bacteroidetes, and Actinobacteria, which is generally consistent with the findings of previous studies in desert soils [38,39,40]. At the phylum level, the abundance of Proteobacteria and Actinobacteria increased in the plastic film-separated treatment group compared to the non-plastic-film-separated treatment group. These bacterial groups are known to help facilitate the uptake of potassium, phosphorus, and other trace nutrients by plant roots [41,42], thereby enhancing nutrient availability. The bacterial community composition did not significantly change between the nylon mesh-separated treatment and the nonseparated and plastic film-separated treatments, indicating that root secretions have a stronger influence on the microbial community structure than direct root contact.

### 3.3. The Impact of Root Interactions on the Metabolite Composition of H. ammodendron Seedling Roots

For primary metabolites, the differentially abundant metabolites between the nylon mesh-separated and plastic film-separated treatments were mainly lipids, lipid-like substances, phenylpropanoids, and polyketides, suggesting their important roles in root secretion interactions. Compared to those in the non-plasma-free treatment, the secretion of lipids and lipid-like substances in *H. ammodendron* seedling roots was greater in the nylon mesh separation treatment, indicating the inhibitory effect of root contact on these compounds. In addition to lipids and lipid-like substances, plastic film separation also increased the secretion of organic acids and their derivatives (mainly carboxylic acids and their derivatives), organic heterocyclic compounds, phenylpropanoids, and polyketides, which may be related to their ability to promote plant growth by recruiting beneficial bacteria [43,44]. Compared to those in the plastic film-separated treatment, the nonangiogenic treatment produced significantly greater amounts of lipids, lipid-like substances, organic acids and their derivatives, organic heterocyclic compounds, phenylpropanoids, and polyketides, indicating that root contact can induce plants to produce more antioxidant and antibacterial compounds to cope with competition pressure. The secretion of these compounds may have an inhibitory effect on the growth and development of surrounding plants, thereby enhancing their own competitive ability.

For secondary metabolites, in *H. ammodendron* seedling roots, the use of nylon mesh separation and plastic film separation led to an increase in the secretion of terpenoid compounds and a decrease in the secretion of alkaloid compounds compared to those in the nonseparated treatment. It has been confirmed that terpenoid compounds and alkaloid compounds play important roles in plant defense [45,46]. For example, terpenoid compounds extracted from *Sphagneticola trilobata* can defend against damage by increasing the activity of defense enzymes such as phenylalanine ammonia-lyase (PAL) [47]. Submerged plants inhibit algal growth by secreting phenolic acids, alkaloids, terpenoids, flavonoids, and other allelochemicals [48]. Therefore, it is speculated that Deacetylisoipecoside and Aurachin D, among the alkaloid compounds secreted by *H. ammodendron* seedlings, play important roles in plant defense.

### 3.4. Effects of Root Metabolites on Soil Nutrients, Plant Traits, and Soil Microbial Communities

The relationships between root metabolites, rhizosphere bacterial communities, plant traits, and soil nutrients demonstrated strong correlations between the root secretions and plant traits. Under plastic film separation, root carbon significantly decreases, while root nitrogen increases significantly, leading to a decrease in terpenoid secretion and an increase in alkaloid secretion, which conforms to the balanced relationship between carbon-based secondary metabolites (such as terpenoids) and nitrogen-based secondary metabolites (such as alkaloids) [49]. An increase in alkaloid substances can enhance plant defense and competitive survival [50,51]. Previous studies have shown that root secretions can improve soil nutrient conditions or serve as signaling molecules in root communication, bringing about positive effects in plant interactions [7,52]. Overall, chemical substances such as 10-deacetylbaccatin III, crocin, swertiamarin, limonin from the terpenoid group, anthragallol from the polyketone group, and O-methylandrocymbine from the alkaloid group exhibit significant positive correlations with the soil nutrients C, N, and P. These compounds are believed to improve soil nutrient availability, enhance nutrient utilization efficiency, and promote plant growth.

There was a significant correlation between terpenoid and alkaloid compounds and the composition of rhizosphere bacterial communities under root–root interactions, as shown in Figure 5. Specifically, terpenoid compounds such as crocin, swertiamarin, limonin, and the polyketone lancerin showed strong positive correlations with the bacterial community, while alkaloid compounds such as aurachin D and deacetyllisoipecoside showed negative correlations. These correlations are particularly strong for Proteobacteria, Actinobacteria, and the genus Pseudomonas. Both the nonseparation treatment and the nylon mesh separation treatment increased the relative abundance of bacteria such as Proteobacteria and Actinobacteria, indicating that the chemical substances secreted by *H. ammodendron* plant roots alter the structure of the soil rhizosphere bacterial community, improving the efficiency of plant utilization of soil moisture, nutrients, and other environmental factors to adapt to biotic stress [53]. In the present study, there were significant positive correlations between soil carbon, nitrogen, and phosphorus levels and between the phyla Proteobacteria and Actinobacteria, as well as between the core terpenoid compounds. Previous literature has shown that terpenoids and phenols are the main allelopathic toxins produced by higher plants, and terpenoid substances secreted by vegetation roots, such as *Medicago sativa*, *Atractylodes lancea*, and *Juncus effusus*, can cause strong autotoxic effects [54,55]. Therefore, we speculate that there may be autotoxicity among *H. ammodendron* seedlings, and crocin, swertiamarin, limonin, and anthragallol may be the main allelochemicals produced by *H. ammodendron* seedlings.

## 4. Materials and Methods

### 4.1. Experimental Design

On 17 May 2022, healthy and similar-sized *H. ammodendron* seedlings were collected from the Ebinur Lake Wetland National Nature Reserve (ELWNR) (44°30′–45°09′ N, 82°36′–83°50′ E; elevation 189 m). The plants were subsequently transplanted into experimental pots. The pots were 29 cm in diameter and 43 cm in height. The plants were filled with a mixture of peat soil, in situ soil, and perlite at a ratio of 2:1:1. The in situ soil was randomly sampled from the surface layer (0–10 cm) of the *H. ammodendron* community in the Ebinur Lake area. After weathering, the mixed soil was passed through a 2 mm sieve to remove debris, after which the soil pH, total nitrogen, total phosphorus, organic carbon, and other nutrient contents were measured.

This experiment included the following three planting methods, aiming to investigate the role of root exudates in plant–plant competition in *H. ammodendron* plants. Two randomly selected plants were planted apart at a distance of 15 cm and at an angle of 180° with respect to the center of the pot. A randomized complete block design was used, and the pots were divided into three groups. The first group received no treatment. The second and third groups were separated underground using 30 μm nylon mesh and plastic film, respectively. The 30 μm nylon mesh allowed chemical and microbial interactions while preventing root penetration, while the plastic film completely blocked the interaction between the roots and the soil (plastic film separation). Each treatment was replicated four times, resulting in a total of 12 pots. All pots were placed in a greenhouse with temperatures ranging from 20–30 °C during the day and night and a relative humidity ranging from 65% to 90%. Watering was performed every six days. Sampling work began in October 2022, and various indicators were measured for *H. ammodendron* seedlings and rhizosphere soil.

### 4.2. Sample Collection and Processing

The plants were carefully removed from the pots and the soil around the roots (about 1–5 cm) was collected in a plastic ziplock bag; then, the soil physico-chemical indicators were determined. Soil pH and electrical conductivity (EC) were determined using a pH meter and a conductivity meter; soil organic carbon (SOC) and total nitrogen (STN) were determined using the potassium dichromate method and the Kjeldahl method [56]; soil nitrate nitrogen (SNN) was determined using the phenoldisulfonic acid colorimetric method, whereas the soil ammonium nitrogen (SAN) was determined using the colorimetric blue indophenol method [57]. The soil available phosphorus (SAP) was determined by the Mo-Sb anti-spectrophotography method [58]. The soil that tightly adhered to the surface of the plant roots (approximately 1–2 mm) was washed with PBS solution (137 mmol/L NaCl, 2.7 mmol/L KCl, 8.5 mmol/L Na_2_HPO_4_, and 1.5 mmol/L KH_2_PO_4_, pH 7.3), collected as rhizosphere soil, placed in a sterilized 50 mL tube, and subsequently stored at −80 °C for the determination of the soil bacterial community structure. Microbial diversity was based on the Illumina NovaSeq sequencing platform and used a two-end sequencing (Paired-End) approach to build small fragment libraries for sequencing. Reads were filtered by splicing, clustering, or denoising, and species annotation and abundance analysis were performed. Primers used 16s rDNA universal primers. The root exudates were collected using a water extraction method [59,60] by soaking for 24 h. The roots were first rinsed with distilled water and then the rinsed root system was filled into a 500 mL measuring cylinder containing 500 mL of distilled water [61,62]; the periphery of the cylinder was covered with tin foil to create a dark environment underground. After 24 h, we removed the plants [63]. The solution inside the cylinder served as the root secretion solution, which was concentrated to 15 mL at 40 °C conditions using a rotary evaporator and stored frozen at −80 °C. Utilizing dry ice for low-temperature transport, it was shipped to Beijing Baimaike Biotechnology Co., Ltd., Beijing, China. for sample testing, where metabolites were identified and classified using liquid chromatography-mass spectrometry (LC-MC). After extraction of the root exudates, the aboveground and underground parts of the plants were separated, the total lengths and total areas of the plant roots were calculated using a WinRHIZO root scanning analysis system (Québec, QC, Canada), and the dry weight and aboveground biomasses of the roots were determined through drying and weighing procedures. The specific root length (area) was calculated by dividing the total root length (area) by the root dry weight. The organic carbon, total nitrogen, and total phosphorus contents of the plants were determined through laboratory chemical experiments. Plant organic carbon (C) was determined by the potassium dichromate dilution heat method. The plant total nitrogen (TN/RTN) was determined by the Kjeldahl method [56]. The plant total phos-phorus (TP/RTN) was determined using a molybdenum antimony anti-colorimetric method [58].

### 4.3. Statistical Analysis

The statistical analysis and data processing were performed in Excel 2019. After checking the normality and homogeneity of variances using Origin 2023, the Kruskal–Wallis sum–rank test was conducted to analyze the data relating to the aboveground and underground growth, morphology, and soil physicochemical properties of *H. ammodendron* seedlings under different separation methods. To evaluate the relationship between variables, we utilized Spearman correlation analysis with the corrplot package in R (version 3.5.3). Additionally, we employed the Mantel test to assess the correlation between different datasets using the linkET package (version 0.0.3).

## 5. Conclusions

In the present study, strong negative interactions were observed among *H. ammodendron* plants, and the mechanism of these interactions was mediated through underground chemical substances and root contact. The mediating effect of underground chemical substances also had a significant impact on root traits (such as specific root length, specific root area, and the root-to-shoot ratio) and soil nutrients such as SOC, STN, and STP. The separation of plant roots by nylon mesh increased the secretion of terpenoids, polyketones, and other compounds as well as specific root traits and soil carbon, nitrogen, and phosphorus contents. When facing root competition, *H. ammodendron* roots significantly increased the secretion of core terpenoid compounds (such as crocin and swertiamarin) and decreased the secretion of alkaloid compounds such as aurachin D to increase the soil nutrient content and enrich the community of deformable bacteria and Acidobacteria in the rhizosphere. This behavior improved the growth environment of *H. ammodendron* plants, inhibited neighboring plants, and thus enhanced competitive advantage. The above conclusion essentially validates our proposed hypothesis that compounds released by the roots of *H. ammodendron* seedlings play a more significant role in competition, particularly compounds such as terpenes, polyketides, and alkaloids. This study provides new insights into intraspecific interactions and coexistence mechanisms among *H. ammodendron* plants in desert regions. In the future, interactions between *H. ammodendron* and other typical desert plants should be considered, to gain a more comprehensive understanding of plant interactions, coexistence mechanisms, and community assembly in ecosystems.

## Figures and Tables

**Table 1:** Correlations of physico-chemical properties of soil at the root perimeter of *H. ammodendron* seedlings. ** indicates a significant correlation at the *p* < 0.01 level; * indicates a significant correlation at the *p* < 0.05 level. The meanings of the letters can be found in Figure 2.

	EC	pH	SOC	STN	STP	SAN	SNN	SAP
EC	1	−0.509	0.695 *	0.819 **	0.437	−0.004	0.280	0.509
pH		1	−0.731 **	−0.714 **	−0.616 *	0.102	−0.156	−0.403
SOC			1	0.850 **	0.800 **	−0.278	0.144	0.598 *
STN				1	0.639 *	0.016	0.202	0.548
STP					1	−0.552	0.403	0.460
SAN						1	−0.014	0.018
SNN							1	0.320
SAP								1

**Table 2:** Types of metabolites in the roots of *H. ammodendron* seedlings under different isolation methods.

No. Root Exduates		D2 vs. N		D2 vs. P		N vs. P		%
		Up	Down	Up	Down	Up	Down	
1	Alkaloids and derivatives	5	0	7	6	2	4	0.92
2	Benzenoids	13	11	41	22	43	17	4.66
3	Homogeneous nonmetal compounds	0	0	0	4	0	0	0.12
4	Hydrocarbon derivatives	0	0	0	0	0	0	0.02
5	Hydrocarbons	2	0	6	0	5	0	0.20
6	Lignans, neolignans, and related compounds	0	1	0	1	1	1	0.35
7	Lipids and lipid-like molecules	77	12	168	58	112	29	13.92
8	Nucleosides, nucleotides, and analogues	7	3	24	24	20	3	2.32
9	Organic acids and derivatives	47	8	74	35	54	17	7.31
10	Organic nitrogen compounds	4	1	4	7	1	0	0.65
11	Organic oxygen compounds	15	3	44	22	41	12	4.91
12	Organohalogen compounds	0	0	1	0	1	0	0.02
13	Organoheterocyclic compounds	43	20	64	41	32	6	5.91
14	Organosulfur compounds	1	0	1	2	1	0	0.10
15	Phenylpropanoids and polyketides	13	1	63	18	66	12	5.66
16	Other	225	38	591	239	486	138	52.91

**Table 3:** Differential changes in KEGG compound classification annotations for secondary metabolites under different isolation methods.

Plant Secondary Metabolites	Up			Down		
	D2 vs. N	D2 vs. P	N vs. P	D2 vs. N	D2 vs. P	N vs. P
Alkaloids derived by amination reactions	2	1				1
Alkaloids derived from lysine		1				
Alkaloids derived from nicotinic acid	1	1				
Alkaloids derived from ornithine						
Alkaloids derived from tryptophan and anthranilic acid	2	6	2	1	2	
Alkaloids derived from tyrosine	1	2	1			
Betalains		2				
Cyanogenic glucosides						
Glucosinolates						
Carotenoids and apocarotenoids		1				
Diterpenoids (C20)		3	1			1
Monoterpenoids (C10)	1	5	3		1	
Sesquiterpenoids (C15)	2	2	2			
Steroids					1	1
Triterpenoids (C30)	1	4	1	1	1	1
Coumarins		3	1			
Monolignols		4	3			
Fatty acids		1			1	
Flavonoids	1	4	2			
Isoflavonoids	2	2				
Anthraquinones	1	2				
Pyrones	1	1	1			

## Data Availability

Data will be made available on request.

## Appendix A

**Table A1:** Number of differentially abundant metabolites between different isolation methods.

Group	Total	Diff	Up	Down
D2 vs. N	4010	358	301	57
D2 vs. P	4010	1182	916	266
N vs. P	4010	824	678	146
